# Roving oddball paradigm elicits sensory gating, frequency sensitivity, and long-latency response in common marmosets

**DOI:** 10.1016/j.ibneur.2021.09.003

**Published:** 2021-09-22

**Authors:** Jamie A. O’Reilly

**Affiliations:** College of Biomedical Engineering, Rangsit University, 52/347 Muang-Ake, Phaholyothin Road, Pathumthani 12000, Thailand

**Keywords:** Mismatch negativity, Predictive coding, Deviance detection, Sensory adaptation, Auditory neurophysiology

## Abstract

Mismatch negativity (MMN) is a candidate biomarker for neuropsychiatric disease. Understanding the extent to which it reflects cognitive deviance-detection or purely sensory processes will assist practitioners in making informed clinical interpretations. This study compares the utility of deviance-detection and sensory-processing theories for describing MMN-like auditory responses of a common marmoset monkey during roving oddball stimulation. The following exploratory analyses were performed on an existing dataset: responses during the transition and repetition sequence of the roving oddball paradigm (standard -> deviant/S1 -> S2 -> S3) were compared; long-latency potentials evoked by deviant stimuli were examined using a double-epoch waveform subtraction; effects of increasing stimulus repetitions on standard and deviant responses were analyzed; and transitions between standard and deviant stimuli were divided into ascending and descending frequency changes to explore contributions of frequency-sensitivity. An enlarged auditory response to deviant stimuli was observed. This decreased exponentially with stimulus repetition, characteristic of sensory gating. A slow positive deflection was viewed over approximately 300–800 ms after the deviant stimulus, which is more difficult to ascribe to afferent sensory mechanisms. When split into ascending and descending frequency transitions, the resulting difference waveforms were disproportionally influenced by descending frequency deviant stimuli. This asymmetry is inconsistent with the general deviance-detection theory of MMN. These findings tentatively suggest that MMN-like responses from common marmosets are predominantly influenced by rapid sensory adaptation and frequency preference of the auditory cortex, while deviance-detection may play a role in long-latency activity.

## 1 Introduction

Human MMN is a negative amplitude deflection between 100 and 250 ms in the difference waveform obtained by subtracting the standard event-related potential (ERP) from the deviant ERP elicited by a passive oddball paradigm (Näätänen et al., 2007). This is widely believed to reflect a process of automatic stimulus discrimination, which has been incorporated into the predictive coding theory of perception, known as deviance-detection (Fitzgerald and Todd, 2020, Ross and Hamm, 2020). In various animal species, MMN-like responses elicited by oddball stimulation are used to model human MMN, with less emphasis placed on polarity or latency range (Featherstone et al., 2018). While the deviance-detection theory of MMN is widely supported throughout the research literature, several findings oppose this interpretation (May and Tiitinen, 2010, O’Reilly, 2021, O’Reilly and O’Reilly, 2021). For example, it is generally accepted that differential adaptation can explain some of the difference between standard and deviant ERPs. Moreover, modulation of intrinsic, obligatory components of the auditory response caused by physical properties of stimuli can also explain some of these MMN-like waveforms (O’Reilly, 2021, O’Reilly and Conway, 2021). With that said, the generative mechanisms of MMN and MMN-like responses are worthy of further examination. Various human neurological conditions reportedly cause abnormal MMN, which is most closely associated with schizophrenia spectrum disorders, spurring calls to develop MMN as a clinical tool for early diagnosis and treatment prognostication (Perez et al., 2017, Schall, 2016, Tada et al., 2019). Expectations that comparable MMN-like deficits can be modelled in animals have also attracted significant research interest (Featherstone et al., 2018). It is thought that studying these animal models can provide insights regarding the underlying generative mechanisms of MMN and its physiology in non-pathological and pathological states. To support these efforts, control sequences are often used in animal studies to provide evidence of whether the observed difference waveforms are primarily influenced by adaptation or deviance-detection (Featherstone et al., 2018). Unfortunately, however, many of these controls suffer from additional confounds, such as contrast gain control (Lohse et al., 2020), that may obstruct direct comparisons of auditory responses elicited during control and oddball paradigms.

Common marmosets (*Callithrix jacchus*) may be better suited than rodents for studying MMN because they are more closely related to humans on the evolutionary tree and have more comparable auditory faculties (Shaheen et al., 2021). The first study demonstrating MMN-like responses in common marmosets included only two monkeys (Komatsu et al., 2015). This is representative of non-human primate neurophysiology research in general, where typically fewer experimental subjects are included compared with rodent or human studies, and data analysis may be performed at the level of a single subject (Fishman and Steinschneider, 2012, Javitt et al., 1996, Shaheen et al., 2021). Limiting subject numbers partly balances the ethical equation, which can be further justified by maximizing insights gained from such experiments. In recognition of this, researchers can opt to share their data openly with the international research community through online repositories such as Neurotycho.org (Nagasaka et al., 2011). Komatsu et al. (2015) used a roving oddball paradigm with different frequency pure-tone auditory stimuli, and uploaded data from one of their monkeys for further research and education. Their published findings identify an MMN-like response between 50 and 120 ms post-stimulus, providing a basis for the development of the common marmoset as a model of MMN. Mechanisms underlying this response were not discussed at length, although experiments to investigate its potential relationship with NMDA receptor function were proposed; research which has since made progress (Komatsu and Ichinohe, 2020). The authors did not express consideration for alternative explanations for the observed MMN-like response, briefly stating that it conveys information regarding violation of a regularity with the auditory input stream (Komatsu et al., 2015), which is generally in-keeping with the prevailing theoretical interpretation of MMN (Fitzgerald and Todd, 2020, Ross and Hamm, 2020).

In the present study, this data from one of the animals in the seminal work on MMN-like responses in common marmosets is revisited to explore some additional factors that may have contributed to this difference waveform. The roving oddball sequence implements physically identical stimuli as both standards and deviants, with the intention of removing the confounding factor of using physically distinct stimuli to derive MMN-like difference waveforms (An et al., 2021, Baldeweg et al., 2004, Baldeweg and Hirsch, 2015, Komatsu et al., 2015). Regrettably this does not account for adaptation or non-linear responses to frequency transitions that may induce amplitude deflections in derived MMN-like waveforms (O’Reilly and O’Reilly, 2021). Hence this report considers whether adaptation, deviance-detection, or frequency-sensitivity is elicited by roving oddball stimulation in the common marmoset. Adaptation and deviance-detection theories of MMN are explored by comparing the effects of stimulus repetitions on both standard and deviant responses. These two competing hypotheses may be contrasted as follows: 1) adaptation may cause the standard response to decrease with increasing repetitions, and 2) deviance-detection may cause the deviant response to increase with increasing numbers of preceding standards. Both may account for observed increases in MMN amplitude with increasing numbers of standards (Sams et al., 1983, Yagcioglu and Ungan, 2008). However, the former would reflect adaptation or habituation (May and Tiitinen, 2010, Ruiz-Martínez et al., 2020), while the latter would reflect the degree of unpredictability or unexpectedness modulating deviance-detection mechanisms (Baldeweg and Hirsch, 2015).

There is evidence from both animals and humans that deviance-detection may occur later than previously considered (Casado-Román et al., 2020, Chen et al., 2015, O’Reilly, 2019, O’Reilly and Angsuwatanakul, 2021, Ruiz-Martínez et al., 2021). As such, the long-latency response to deviant stimuli was extracted using a double-epoch waveform subtraction (O’Reilly, 2019). This may be a particularly relevant latency range, given that laterERP are believed to reflect higher-order cognitive processes, whereas earlier components are considered to reflect afferent sensory processes (Luck and Kappenman, 2011). Influence of frequency-sensitivity was also examined by first establishing the preferred frequency of tissue recorded by electrodes closest to the auditory cortex, then comparing the effects of ascending versus descending frequency transitions on the resulting MMN-like response. Enhancement of the MMN-like response by transitions towards the preferred frequency might suggest that frequency sensitivity plays a role in difference waveform morphology; no difference between ascending and descending frequency transitions would suggest that general deviance-detection mechanisms predominate.

## 2 Methods

### 2.1 Data

The data analyzed in this study originated from previous research (Komatsu et al., 2015), in which epidural electrocorticography (ECoG) was recorded from two male common marmoset monkeys while they were awake. Experimental procedures were conducted in accordance with the National Institutes of Health Guidelines for the Care and Use of Laboratory Animals and approved by the RIKEN Ethical Committee (Komatsu et al., 2015). Data from one of these monkeys ("Fr") is publically available via an online repository (Nagasaka et al., 2011) for further research and education. During recordings, ECoG signals were referenced to an occipital electrode (Komatsu et al., 2015). Thirty-two electrode channels were inspected and one ("Ch25") displayed particularly robust auditory evoked responses, as identified by Komatsu et al. (2015). The following analysis concentrates on this temporal electrode site, which presumably reflects activity from auditory processing areas of the cortex.

### 2.2 Auditory stimulation

**Fig. 1:**
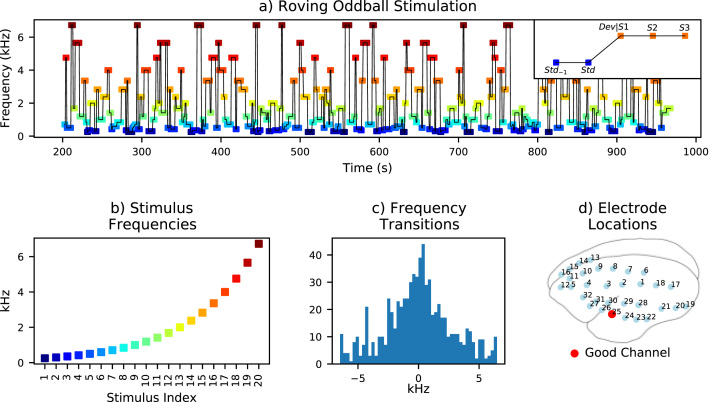
Auditory stimulation and recording electrode setup. The roving oddball paradigm (a) was deployed with 20 different frequency stimuli (b); the inset in (a) shows a transitional sequence between different frequency tone-trains, identifying the stimulus before the standard (Std_−1_), standard (Std), deviant (Dev/S1), and two subsequent stimuli (S2 and S3). Ascending and descending frequency transitions (c) were reasonably well-balanced across the experimental protocol. From 32 ECoG recording channels (d), one good channel (25) located on the temporal lobe, presumably closest to the auditory cortex, exhibited particularly robust auditory evoked potentials and was selected for further analysis (see Fig. S1 for all channels).

Identical sessions were performed three times over three different days, and the data recorded during these sessions was pooled for analysis. The roving oddball paradigm consisted of 240 stimulus trains, each with one of 20 different frequency tones repeated 3, 5, or 11 times, before starting a new tone-train with a different frequency. The last stimulus in each tone-train was considered to be the standard and the first stimulus in each new (different frequency) tone-train was considered to be the deviant. Tone-trains were balanced across frequencies, with 12 of each, and number of repetitions, with 4 of each for each frequency. This produced a total of 20 × 4 × (3 + 5 + 11) = 1520 pure-tone auditory stimuli per session. Stimuli were 64 ms in duration with 7 ms rise and fall times, presented with constant stimulus onset asynchrony (SOA) of 503 ms. Stimulus frequencies ranged from 250 Hz to 6.727 kHz in quarter-octave increments, and the transitions between different frequency stimulus trains were fairly well balanced, as illustrated in Fig. 1. Descending and ascending frequency transitions respectively accounted for 49.03% and 50.97% of changes between different frequency tone-trains in the roving oddball sequence. These auditory stimuli reportedly had an average sound pressure level of 60 dB at the location of the monkey's ear (Komatsu et al., 2015).

### 2.3 ERP analysis

#### 2.3.1 Preprocessing

Channel-average re-referencing was performed and signals were band-pass filtered between 0.1 and 30 Hz, in agreement with (Komatsu et al., 2015). Segments of ECoG data used for computing ERPs were extracted from 100 ms pre-stimulus with average baseline correction. Analysis was performed on channel 25, which demonstrated reliable auditory evoked responses (see Fig. S1 for comparison between channels), and no epochs were rejected from inclusion in the ERP calculation.

#### 2.3.2 Stimulus repetition effects

The key repeating sequence of the roving oddball paradigm consists of the transition between the final stimulus of one tone-train (referred to as the standard) and the first stimulus of the next tone-train (referred to as the deviant). MMN-like responses are obtained by subtracting the standard ERP from the deviant ERP. Typically only standard and deviant responses are analyzed from roving oddball stimulation (An et al., 2021, Baldeweg et al., 2004, Baldeweg and Hirsch, 2015, Komatsu et al., 2015), and responses to other stimuli or directionality of the physical parameter being manipulated are not considered. The present study addresses these factors that may have been overlooked previously.

**Fig. 2:**
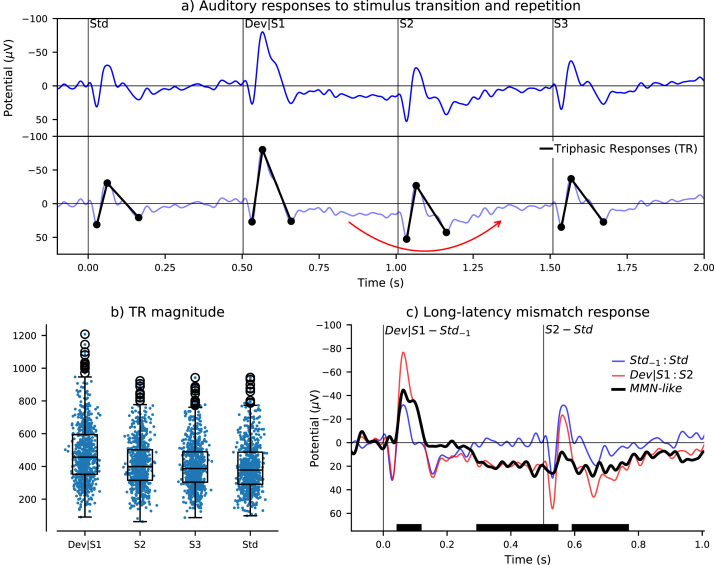
Response to key repeating sequence of the roving oddball paradigm. Averaged responses from 717 ECoG segments (a, upper) recorded during consecutively presented stimuli at the transition between one stimulus train and the next (Std, Dev/S1, S2, and S3) were quantified using triphasic response (TR) magnitude (a, lower). Quantification of this triphasic response (b) indicates that the deviant first stimulus produced a larger response, whereas subsequent responses were similar. The double-epoch subtraction highlighted a long-latency mismatch response (c), annotated with a red arrow in the lower panel of (a); solid black bars at the bottom of the plot represent statistically significant regions of difference in the MMN-like waveform (FDR-corrected p < 0.01). (For interpretation of the references to colour in this figure legend, the reader is referred to the web version of this article.)

Auditory responses to stimuli immediately before and after the deviant were investigated. The minimum number of repetitions for each stimulus was 3, hence the four-stimulus sequence of standard -> deviant/S1 -> S2 -> S3 was repeated 239 times in each recording session, producing 717 epochs in total. Consecutive stimuli in tone-trains are denoted S1, S2, S3, and so on, while the first and last stimuli may also be denoted as deviant and standard, respectively. This analysis is presented in Fig. 2. One third of the epochs included among standard trials were shared with S3; the other two thirds were from S5 and S11 trials.

**Fig. 3:**
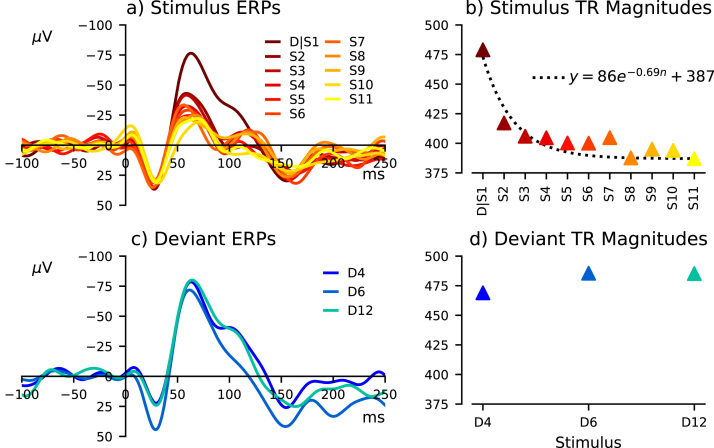
The effects of stimulus repetitions on the auditory responses to identical sitmuli (a,b) and deviant (c,d) stimuli. Repeated identical stimuli are labelled D/S1 to S11, representing the first to the eleventh consecutive stimulus presentations; D/S1 to S3 were computed from 720 epochs, S4 to S5 were computed from 480 epochs, and S6 to S11 were computed from 240 epochs. The average triphasic response (TR) magnitude of repeated stimuli decreased exponentially, as shown in (b). Deviant stimuli presented after 3, 5, and 11 repetitions of a proceeding stimulus are denoted D4, D6, and D12, respectively. The number of preceding stimuli does not appear to influence the deviant response.

The number of consecutive identical stimulus repetitions preceding an unexpected switch to a physically different stimulus has previously been interpreted to reflect the MMN memory trace effect (Baldeweg et al., 2004, Baldeweg and Hirsch, 2015), which may be described in terms of a perceptual predictive model. More standards presumably instill greater confidence in the predictive model, thus more exaggerated prediction errors would be elicited by deviant stimuli. Adaptation and deviance-detection theories can be evaluated by comparing the influence of stimulus repetitions on auditory responses to standard and deviant stimuli. Adaptation would decrease the response to repeated standard stimuli, while deviance-detection would increase the response to deviant stimuli. Thereby it may be possible to dissociate adaptation from deviance-detection components of the MMN-like response. ERPs elicited by stimulus repetitions are labelled D/S1 to S11; there were 720 epochs of S1 to S3, 480 epochs of S4 to S5, and 240 epochs of S6 to S11. ERPs produced by deviant stimuli after 3, 5, or 11 repetitions of a preceding stimulus are labelled D4, D6, and D12, respectively. This analysis is shown in Fig. 3.

#### 2.3.3 Long latency response

The deviant stimulus appeared to induce a slow positive amplitude component over approximately 300–800 ms after stimulus onset (see Fig. 2a). This exceeded the typical window of analysis for MMN-like difference waveforms, which is limited by the inter-stimulus interval. As such, the double-epoch waveform subtraction (O’Reilly, 2019, O’Reilly and Angsuwatanakul, 2021) was applied to analyze this long-latency feature. This consisted of subtracting consecutive Std_−1_ and Std responses from consecutive Dev/S1 and S2 responses (i.e. Dev/S1:S2 minus Std_−1_:Std), as shown in Fig. 2c. One third of Std_−1_ and Std epochs were shared with S2 and S3, respectively, because one third of the tone-trains consisted of three stimulus repetitions. The other two thirds of Std_−1_ epochs came from S4 and S10 trials, while the other two thirds of Std epochs came from S5 and S11 trials.

#### 2.3.4 Frequency sensitivity

**Fig. 4:**
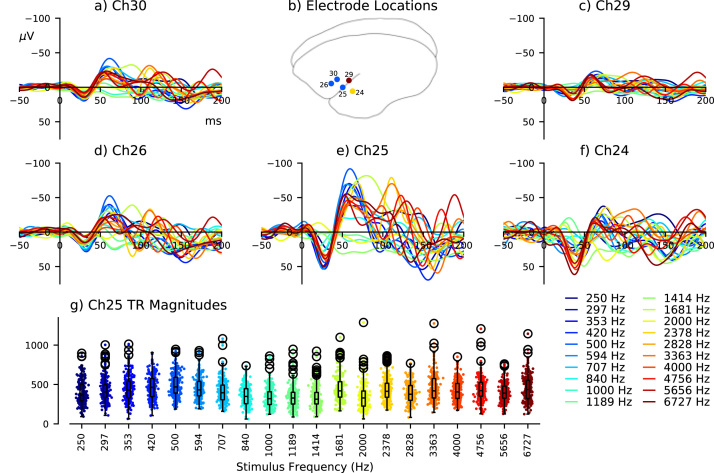
Effects of stimulus frequency. Electrodes surrounding the auditory cortex (b) are colored according to the frequency eliciting maximum peak-to-peak ERP amplitude. Channels 30 (a), 26 (d), and 25 (e) were most sensitive to 500 Hz tones; channels 29 (c) and 24 (f) were most sensitive to 6727 Hz and 2378 Hz tones, respectively. Triphasic response (TR) magnitudes measured from channel 25 in response to different frequency stimuli are plotted in (g), and correspond with maximum ERP amplitude in response to 500 Hz tones. Differences between channels may suggest that cortical ERPs are influenced by frequency-sensitive tissue responses proximal to the recording electrode site.

The marmoset auditory system is known to exhibit tonotopy from the cochlea to the cortex (Tani et al., 2018). Therefore it is reasonable to consider that different frequency stimuli could induce differential auditory responses. The roving oddball paradigm is designed to avoid this confound, although it is uncertain whether ascending or descending frequency transitions engender preferentiality of the auditory response. To evaluate this, evoked responses from different frequency stimuli were analyzed (n = 228 for each frequency), as shown in Fig. 4. This analysis included electrodes surrounding the auditory cortex, providing some insight regarding the spatial extent of this frequency-sensitivity. Transitions between tone-trains were also separated into instances of ascending (n = 366) and descending frequency (n = 351) to examine any effect of this frequency-sensitivity on corresponding MMN-like responses.

#### 2.3.5 Difference waveforms

**Fig. 5:**
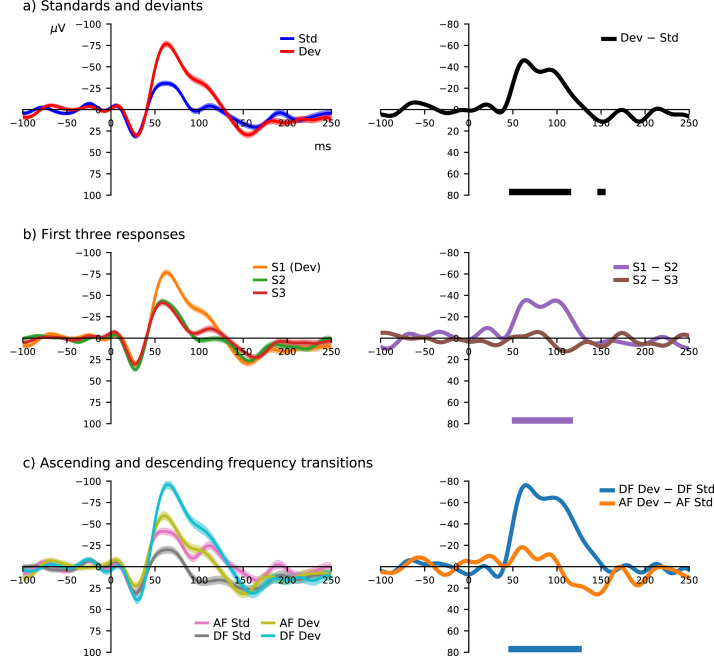
Comparison of difference waveforms. Conventional MMN (a), first three stimuli responses (b), and ascending vs. descending frequency transition deviances (c) are shown. The top row replicates the analysis of (Komatsu et al., 2015). The middle row illustrates differences between responses to first (Dev/S1), second (S2), and third (S3) stimuli in a tone train; deviant/first-stimulus response enhancement is essentially absent from the second stimulus, which is almost identical to the third. The bottom row presents the difference between ascending (AF; Std < Dev) and descending (DF; Std > Dev) frequency transitions, highlighting preferential enlargment of the response to descending frequency deviant stimuli. ERP waveforms on the left are plotted as mean with shaded standard error of the mean. Statistically significant differences (FDR-corrected p < 0.01) are annotated with colour-coded bars below respective difference waveforms on the right.

Conventional MMN-like difference waveforms were analyzed, in agreement with (Komatsu et al., 2015), by subtracting the standard ERP from the deviant ERP. In addition to this, difference waves between first, second, and third stimuli in each tone-train were computed as follows: S1 (deviant) minus S2, and S2 minus S3. These comparisons were made to understand the importance of the standard condition (i.e. a preceding different frequency tone) versus consecutive presentations of identical stimuli. Standards and deviants from ascending and descending frequency transitions were also analyzed to establish whether these dynamics have a dissociable influence on the observed MMN-like response. All of these MMN-like difference waveforms are plotted in Fig. 5.

#### 2.3.6 Triphasic response magnitude

Triphasic response magnitude was used to quantify general responsiveness to auditory stimuli, and consisted of the absolute distance between three points of the evoked waveform; a positive peak from 0 to 50 ms, negative peak from 50 and 100 ms, and second positive peak from 100 and 200 ms post-stimulus-onset. This is illustrated in the lower panel of Fig. 2(a). For each stimulus, triphasic response magnitude was computed from each segment of ECoG recorded from channel 25. Resulting data are plotted in Figs. 2(b), 3(b), 3(d), and 4(g). Difference waveforms were evaluated by comparing their two contributing sets of ERP trials at every time point, as described below, not using this triphasic response measurement.

### 2.4 Statistical analysis

Single-trial triphasic response magnitudes were analyzed. Normality of this data could not be assumed. As such, nonparametric statistical tests were performed. Standard, deviant/S1, S2, and S3 data shown in Fig. 2(b) were compared with pairwise Wilcoxon rank-sum tests with Bonferroni corrections for multiple comparisons. The effects of consecutive stimulus repetitions on standards (S1 to S11) and deviants (D4, D6, and D12) were evaluated with Kruskal-Wallis H-tests. Given that the assumption of independence between samples was violated, as all of the data was obtained from one subject, the alpha value was selected to be 0.01.

To assess whether non-zero amplitudes in MMN-like difference waveforms were statistically significant, the corresponding ERP trials were compared at every time-point from 0 to 300 ms post-stimulus-onset using Wilcoxon rank-sum tests with false discovery rate (FDR) corrections (Benjamini and Yekutieli, 2001). Statistically significant results with corrected p-values below 0.01 are annotated below the respective difference waveforms in Fig. 5. This approach was also used for long-latency responses over an extended latency range of 0–1000 ms, as shown in Fig. 2(c).

It is important to acknowledge that this analysis has been performed on single-subject data. Results must therefore be treated as preliminary, not necessarily generalizable, and subject to further confirmation.

### 2.5 Software

Python 3.7.2 and the following modules were used: MNE-Python 0.21.2, Scipy 1.4.1, Numpy 1.19.5, and Matplotlib 3.3.3. Access to the code for replicating this analysis can be obtained by requesting from the author.

## 3 Results

The averaged auditory evoked response to the key repeating sequence of the roving oddball paradigm is plotted in Fig. 2(a). The first stimulus in the new frequency tone-train (Dev/S1) clearly displays an enlarged response compared with both the preceding, different-frequency standard (Std), and the following same-frequency stimulus (S2). Using triphasic response magnitude to quantify these evoked waveforms, this deviant response enhancement was found to be statistically significant [Dev/S1 vs. S2 t(716) = 6.749, p = 8.919 × 10^−11^; Dev/S1 vs. S3 t(716) = 8.249, p = 9.618 × 10^−16^; Dev/S1 vs. Std t(716) = 9.334, p = 6.099 × 10^−20^; Bonferroni-corrected p-values]. Differences between responses to subsequent stimuli and the standard were not statistically significant, although a pattern towards gradually decreasing amplitude was apparent, as shown in Fig. 2(b) [S2 vs. S3 t(716) = 1.653, p = 0.590; S2 vs. Std t(716) = 2.909, p = 0.022; S3 vs. Std t(716) = 1.277, p = 1.000; Bonferroni-corrected p-values]. It should be noted, however, that because tone-trains consisted of either 3, 5, or 11 repetitions, one third of the standard epochs were also S3 epochs, which partly explains the S3 vs. Std result.

Perhaps a more subtle observation from the waveform presented in Fig. 2(a) is the long-latency positive amplitude deflection following presentation of the deviant stimulus and extending through approximately 800–1300 ms (i.e. 300–800 ms post deviant-stimulus-onset). This long-latency response was examined using a double-epoch subtraction (O’Reilly, 2019, O’Reilly and Angsuwatanakul, 2021), which is shown in Fig. 2(c). Both the enlarged early onset response and this long-latency feature were found to exceed the threshold for statistical significance. All stimuli evoked morphologically comparable onset responses, albeit that the deviant response had greater amplitude, whereas this long-latency feature only followed the deviant stimulus. It is therefore more difficult to ascribe this part of the MMN-like response to modulation of intrinsic sensory mechanisms.

The effects of stimulus repetition on auditory responses are shown in Fig. 3. Increasing numbers of repetitions appear to cause a progressive reduction of the ERP amplitude over approximately 50–150 ms post-stimulus-onset. Triphasic response magnitudes measured from Dev/S1 to S11 epochs accordingly illustrated an exponentially decreasing characteristic, with a statistically significant difference between them [H(10) = 146.196, p = 2.256 × 10^−26^]. In contrast, increasing numbers of identical stimulus repetitions preceding a different (deviant) stimulus did not have such a pronounced effect on triphasic response magnitudes measured from D4, D6 or D12 epochs [H(2) = 1.297, p = 0.523].

Auditory responses elicited by different frequency stimuli are displayed in Fig. 4. This analysis includes data from four electrodes surrounding the good channel identified by Komatsu et al. (2015). Channel 25 clearly exhibits the largest auditory evoked responses, which appear to demonstrate a preference towards lower frequency, 500 Hz tones. Anterosuperior electrodes (Ch26 and Ch30) also displayed their largest response to 500 Hz tones, whereas posterosuperior (Ch29) and posterior (Ch24) channels were maximally stimulated by 6727 Hz and 2378 Hz tones, respectively. Analysis of triphasic response magnitudes in Fig. 4(g) also suggests that the cortical tissue closest to the Ch25 electrode responded preferentially to pure tones of 500 Hz: this observation is relevant for interpreting differences between ascending and descending frequency transitions in the roving oddball paradigm.

Auditory ERPs and their corresponding difference waves are plotted on the left and right sides of Fig. 5, respectively. In Fig. 5(a), the MMN-like waveform produced by subtracting the standard ERP from the Dev/S1 ERP agrees with those previously reported (Komatsu et al., 2015). A similar difference waveform may be derived by subtracting the second stimulus, S2 ERP, from the Dev/S1 ERP, as shown in Fig. 5(b); in contrast, there is no significant difference between second and third stimuli ERPs (S2 minus S3). This indicates that there are negligible difference between the deviant minus standard and deviant minus S2 MMN-like responses. Transitions between repeated tone sequences are separated into instances of ascending (AF) and descending frequency (DF) in Fig. 5(c). Here the DF MMN-like response is large and statistically significant across approximately 50–150 ms, in contrast with the AF MMN-like response. This suggests that frequency sensitivity influences the difference between standard and deviant ERP responses during roving oddball stimulation.

## 4 Discussion

Magnitudes of auditory responses to consecutive stimuli in tone-trains decreased exponentially (Fig. 2, Fig. 3). The first stimulus response (Dev/S1) was much larger, while subsequent responses did not differ significantly from the standard. Although responses after the deviant were not substantially different, there was a perceptible amplitude reduction, which illustrates an exponential decay characteristic (Fig. 3b). Interpretation of deviant response enlargement can be debated, with some perhaps arguing that it represents a prediction error signal (An et al., 2021), others that it reflects absence of stimulus-specific adaptation (May and Tiitinen, 2010), and yet others that it reflects a combination of physical sensitivities and general adaptation of the auditory system (O’Reilly and Conway, 2021, O’Reilly and O’Reilly, 2021). The Dev/S1 minus S2 difference waveform (Fig. 5b) strongly resembles the classic deviant minus standard MMN-like response (Fig. 5a). This suggests that the response to a novel frequency tone becomes essentially equivalent to the standard response after a single presentation, which could be interpreted either as rapid cortical adaptation (Wright et al., 2021) or rapid updating of the predictive model. Some accounts of MMN generation state that several repetitions of a stimulus are required to form a reliable sensory-memory trace/predictive model (Näätänen et al., 2007) whereas others indicate that MMN can be elicited after presentation of a single stimulus (Sams et al., 1984, Yagcioglu and Ungan, 2008); if the mechanisms of MMN generation are related to sensory-memory violation and prediction error signaling, the large decrease in response amplitude between the Dev/S1 and S2 supports the latter position, that a single stimulus is sufficient to update the predictive model. However, these observations also bear a striking resemblance to sensory gating studies, where acoustic stimuli are presented in pairs (Kisley et al., 2004), suggesting that adaptation may be a more fitting description.

Fig. 3(a) and (b) shows that stimulus repetition progressively reduces standard ERP amplitudes across 50–150 ms. This latency range overlaps the MMN-like response shown in Fig. 5 (also identified by Komatsu et al., 2015). As shown in Fig. 3(c) and (d), increasing standard repetitions before a change in tone frequency did not have pronounced effect on deviant (D4, D6, or D12) ERP amplitudes during this latency range. This contrast suggests that the MMN memory trace effect (Baldeweg et al., 2004, Baldeweg and Hirsch, 2015) is caused by adaptation to the standard, opposed to increased responsiveness to deviant stimuli. In view of these findings, perhaps deviance-detection ought to be redefined simply as a difference between states of adaptation. This might be an unsatisfactory compromise for proponents of the deviance-detection theory, who consider deviance-detection to involve distinct memory-based processes that are fundamentally different from sensory adaptation (Näätänen et al., 2005). Therefore resolving the debate between these competing hypotheses as a matter of semantics is unlikely to be sustainable. With more potential confounds coming to light regarding adaptive sensory processes in response to the physical properties of auditory stimulation (Lohse et al., 2020, O’Reilly, 2021), the general deviance-detection theory risks becoming untenable, despite efforts to enshrine it into the definition of a "genuine" MMN-like response (Featherstone et al., 2018). However, there was a distinct feature of deviant-evoked electrophysiology that was absent from the response to repeated standard stimuli, presumably representing the activity of neural mechanisms beyond afferent sensory processing, that could potentially lend support for this somewhat precarious-looking theory.

A long-latency component of the MMN-like difference waveform emerges downstream from the enhanced auditory response to deviant stimuli: visible in Fig. 2(a) and analyzed in Fig. 2(c). This relatively slow, positive amplitude component is apparent from approximately 300 to 800 ms post deviant-stimulus-onset, and was not observed in response to other stimuli. This is similar to findings in anaesthetized rodents that display responses to deviant stimuli extending over a comparable time-course (Casado-Román et al., 2020, Chen et al., 2015, O’Reilly, 2019, O’Reilly and Angsuwatanakul, 2021, Ruusuvirta et al., 1998), and perhaps also to recent observations from conscious humans (Ruiz-Martínez et al., 2021). In urethane-anaesthetized rats, this long-latency activity has been interpreted as evidence for prediction error signaling related to automatic deviance-detection (Casado-Román et al., 2020). In urethane-anaesthetized mice these waveform features have also been reported to satisfy some of the requirements for a "genuine" MMN-like response (Harms et al., 2016, O’Reilly, 2019, O’Reilly and Angsuwatanakul, 2021); although they were also elicited by deviant-alone control paradigm stimuli, and not by lower intensity oddballs (Casado-Román et al., 2020, O’Reilly, 2019). Long-latency single-unit spikes triggered after target stimuli in a behavioral task have also recently been observed from the inferior colliculus of awake marmosets, indicating that this long-latency MMN-like response could involve subcortical communication (Shaheen et al., 2021). The occurrence of deviant response enhancement followed by long-latency activity might be speculatively described as a marmoset analogue of the proposed human MMN-P3a-reorienting negativity reaction to environmentally salient sounds (Horváth et al., 2008). However, the marmoset MMN-like response from 50 m to 150 ms overlaps with obligatory sensory components, which are clearly influenced by adaptation, whereas the long-latency portion does not appear to resemble an obligatory sensory component, thus is arguably more likely to reflect deviance-detection.

One third of epochs comprising Std_−1_ and Std were shared with S2 and S3, respectively. This means that one third of the long-latency mismatch response shown in Fig. 2(c) was derived by subtracting conjoined S2:S3 epochs from Dev/S1:S2 epochs. This again raises the question of whether the response immediately after a deviant can be considered the same as a standard. Looking at Fig. 5(a) and (b), it appears that Dev/S1 minus Std and Dev/S1 minus S2 difference waveforms are almost indistinguishable. Moreover, Fig. 3(a) and (b) shows that the response to a repeated stimulus decreases the most after its initial presentation, with smaller amplitude reductions thereafter. Taken together, this evidence supports the notion that a stimulus repeated once becomes approximately equivalent to a stimulus that has been repeated consecutively multiple times (Sams et al., 1984, Yagcioglu and Ungan, 2008). Therefore this potential confound does not implicitly alter the interpretation of the long-latency mismatch response being related to some form of deviance-detection.

Curiously, in Fig. 3(a), negative amplitude changes in the ERP waveform appear to begin before stimulus onset in trials preceded by at least eight consecutive repetitions. Baseline amplitude fluctuations normally occur due to random variations in biological and non-biological physical processes, and have an inverse relationship with the number of trials used to compute the ERP. Baseline activity can also be affected by consistent overlap in evoked neural activity between consecutive trials (Luck, 2014). However, small early amplitude changes observed from S9 to S11 responses in Fig. 3(a) emerge just slightly before stimulus onset, discounting the likelihood of the source extending from previous trials. Interestingly, these amplitude changes are of opposite polarity to the larger, more consistent positive deflection that peaks at approximately 25 ms post-stimulus-onset. Perhaps this reflects preparatory or entrained activity following repetitive monotonic auditory stimulation, which could potentially lead to an omission response if an expected stimulus was omitted (Horváth et al., 2010). Entrainment of auditory cortex neurons at comparable frequencies provides a neurophysiological mechanism that could feasibly account for this observation (O’Connell et al., 2015). Exploring the relationship between auditory neuron entrainment and omission responses may be a promising direction for future research that seeks to examine the neurophysiological basis for omission MMN.

Spectral preference of the auditory response was examined by visualizing ERPs evoked by different frequency stimuli and calculating their triphasic response magnitudes. Data shown in Fig. 4 indicates that auditory responses from channel 25 were larger for lower frequency stimuli, with the largest response to 500 Hz tones. This pattern does not coincide with the marmoset audiogram (Osmanski and Wang, 2011), which exhibits lower hearing thresholds for tone frequencies towards the higher end of those used in this experiment (≈7 kHz). This may refute the suggestion that spectral hearing sensitivity is responsible for overall auditory response amplitudes (O’Reilly and Conway, 2021), or alternatively, perhaps the recording electrode was located nearby a low-frequency representation of the marmoset auditory cortex (see Fig. S2 from Komatsu et al. 2015), which is known to consist of multiple tonotopic subdivisions (Tani et al., 2018). Auditory responses observed from other channels provide some evidence to support the latter explanation, although electrode spacing is too large to provide a more comprehensive analysis of tonotopy. A study with very similar methods demonstrated sensitivity to different frequency tones in different subjects (Komatsu and Ichinohe, 2020), which may be accounted for by the precise location of recording electrodes in relation to tonotopically sensitive regions of the auditory cortex. Analysis of auditory responses to ascending versus descending frequency transitions in the roving oddball sequence indicates that this plays a central role in shaping MMN-like response morphology. Enlarged responses to descending frequency transitions clearly dominated the MMN-like difference waveform from 50 m to 150 ms, as shown in Fig. 5(c), indicating that both auditory ERPs and MMN-like responses observed here from common marmosets are strongly influenced by frequency-sensitivity. This agrees with previous research that did not find evidence for deviance-detection mechanisms in macaque monkeys (Fishman and Steinschneider, 2012).

The effects of deviant frequency stimuli within the broader context of constant or variable standard frequency tones was recently explored by Schröger and Roeber (2021). They observed that MMN was absent when both higher and lower frequency tones (900 Hz and 1100 Hz) were presented as standards, and deviant frequency tones were midway between the two (1000 Hz). The evoked responses to equal higher and lower, relatively close frequency standards, when averaged together, approximately cancelled out that of the deviant frequency stimulus, producing an MMN difference waveform without statistically significant deflections. This was interpreted to reflect difficulties in stochastic encoding of stimulus probabilities within the hypothesized predictive model of the auditory environment; although could arguably be explained on the basis that the intrinsic auditory response is sensitive to stimulus frequencies. Results of the present study suggest that asymmetry of auditory responses at the level of epidural field-potentials is reflected in the MMN-like response of common marmosets. This does not support the existence of a predictive model, which would presumably generalize to ascending and descending frequency transitions. Disproportionate influence of descending frequency transitions on the MMN-like response indicates that local frequency-sensitivity of tissue proximal to the recording electrode site is responsible for significant differences between standard and deviant stimuli (Fig. 5c). In contrast, scalp-recorded potentials may be less susceptible to regional tonotopic differences and more representative of spectral hearing sensitivity though volume conduction.

The principle limitation of this study is that it was conducted on data from a single animal. Unfortunately this limitation was inevitable, because data from the other monkey in the Komatsu et al. (2015) study is not openly available. As such, these results may be considered preliminary, and statistically significant findings cannot be presumed to generalize to the larger population. Furthermore, a single electrode channel was the focus of analysis. Presumably this channel was located closest to the auditory cortex, given that it demonstrated the most reliable auditory evoked responses. The same channel was also the focus of analysis performed by Komatsu et al. (2015), ensuring some degree of comparability. Despite these limitations, the results of this study challenge the assumption that MMN-like responses in common marmosets are caused by general deviance-detection mechanisms, suggesting that adaptation and frequency-sensitivity play a more pivotal role than previously recognized.

## 5 Conclusions

An enlarged auditory response to deviant stimuli was evoked by roving oddball stimulation. The amplitude of this enhanced response decreased exponentially with stimulus repetition, characteristic of sensory adaptation. Additionally, auditory responses to ascending and descending frequency transitions were not equivalent, suggesting that they can vary depending on the local tissue response summated at the recording electrode site. Deviant stimuli also elicited a long-latency MMN-like response that might be linked to deviance-detection mechanisms, as this was unlike other sensory responses. Overall, this emphasizes the need to consider alternative explanations for differences between standard and deviant responses in the roving oddball paradigm that account for known mechanisms of sensory neurophysiology. However, it is important to acknowledge that this analysis has been performed on data from a single marmoset monkey. As such, generalizability of these findings cannot be guaranteed, and these tentative conclusions require further confirmation.

## Funding statement

This study did not receive any funding from public, private, or not-for-profit agencies.

## CRediT authorship contribution statement

**Jamie A. O'Reilly:** Conceptualization, Data curation, Formal analysis, Methodology, Software, Visualization, Writing – original draft, Writing – review & editing.

## Data Availability

The data used in the study originated from Komatsu et al. (2015) and is available from Neurotycho.org (Nagasaka et al., 2011)
