# Supplementary figures and images for: Roving oddball paradigm elicits sensory gating, frequency sensitivity, and long-latency response in common marmosets
Source: IBRO Neurosci Rep. 2021 Sep 22;11:128–36. doi: 10.1016/j.ibneur.2021.09.003 (PMC8482433; doi:10.1016/j.ibneur.2021.09.003)

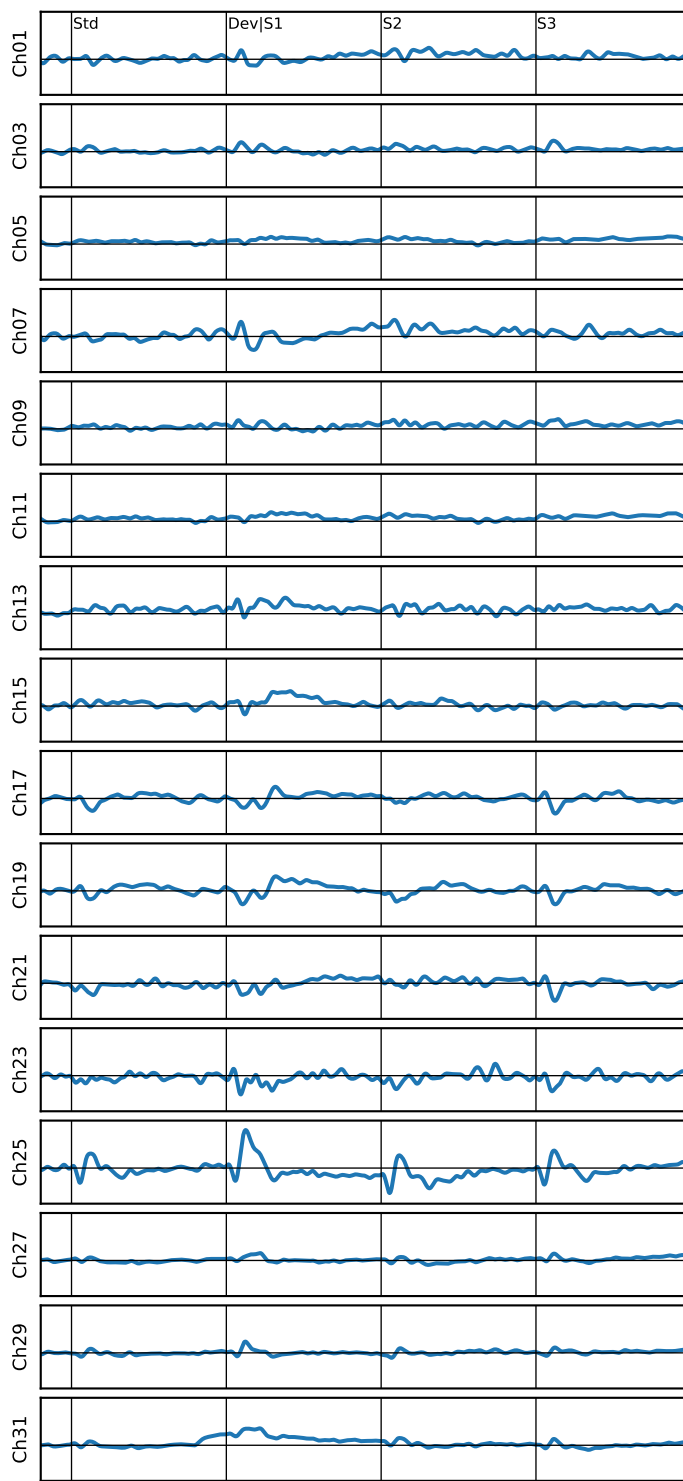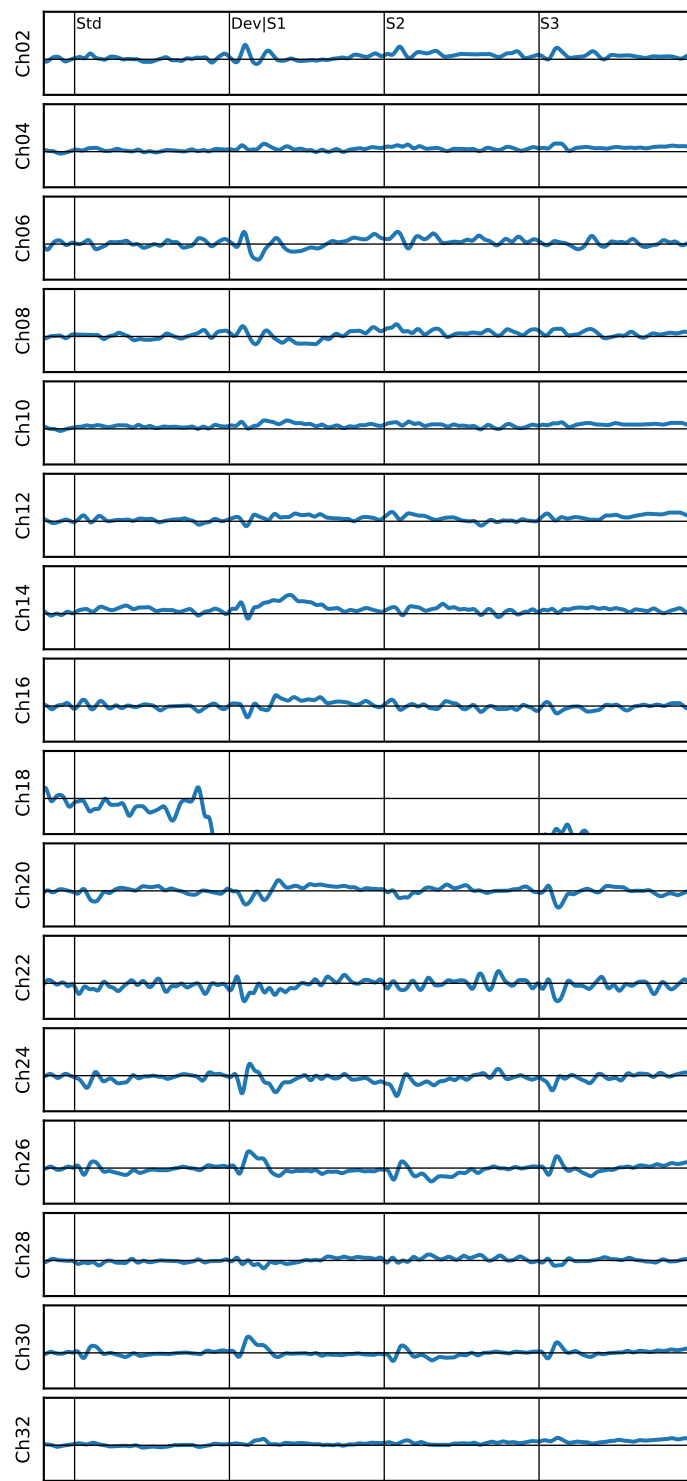

Supplement: Supplementary file 1 — Supplementary material. [file mmc1.pdf]
